# Chronic kidney disease in the context of multimorbidity patterns: the role of physical performance: The screening for CKD among older people across Europe (SCOPE) study

**DOI:** 10.1186/s12877-020-01696-4

**Published:** 2020-10-02

**Authors:** Andrea Corsonello, Paolo Fabbietti, Francesc Formiga, Rafael Moreno-Gonzalez, Lisanne Tap, Francesco Mattace-Raso, Regina Roller-Wirnsberger, Gerhard Wirnsberger, Johan Ärnlöv, Axel C. Carlsson, Christian Weingart, Ellen Freiberger, Tomasz Kostka, Agnieszka Guligowska, Pedro Gil, Sara Lainez Martinez, Itshak Melzer, Ilan Yehoshua, Fabrizia Lattanzio, on behalf of SCOPE investigators, Fabrizia Lattanzio, Andrea Corsonello, Silvia Bustacchini, Silvia Bolognini, Paola D’Ascoli, Raffaella Moresi, Giuseppina Di Stefano, Cinzia Giammarchi, Anna Rita Bonfigli, Roberta Galeazzi, Federica Lenci, Stefano Della Bella, Enrico Bordoni, Mauro Provinciali, Robertina Giacconi, Cinzia Giuli, Demetrio Postacchini, Sabrina Garasto, Annalisa Cozza, Francesco Guarasci, Sonia D’Alia, Romano Firmani, Moreno Nacciariti, Mirko Di Rosa, Paolo Fabbietti, Gerhard Hubert Wirnsberger, Regina Elisabeth Roller-Wirnsberger, Carolin Herzog, Sonja Lindner, Francesco Mattace-Raso, Lisanne Tap, Gijsbertus Ziere, Jeannette Goudzwaard, Tomasz Kostka, Agnieszka Guligowska, Łukasz Kroc, Bartłomiej K. Sołtysik, Małgorzata Pigłowska, Agnieszka Wójcik, Zuzanna Chrząstek, Natalia Sosowska, Anna Telążka, Joanna Kostka, Elizaveta Fife, Katarzyna Smyj, Kinga Zel, Rada Artzi-Medvedik, Yehudit Melzer, Mark Clarfield, Itshak Melzer, Ilan Yehoshua, Francesc Formiga, Rafael Moreno-González, Xavier Corbella, Yurema Martínez, Carolina Polo, Josep Maria Cruzado, Pedro Gil Gregorio, Sara Laínez Martínez, Mónica González Alonso, Jose A. Herrero Calvo, Fernando Tornero Molina, Lara Guardado Fuentes, Pamela Carrillo García, María Mombiedro Pérez, Alexandra Renz, Susanne Muck, Stephan Theobaldy, Andreas Bekmann, Revekka Kaltsa, Sabine Britting, Robert Kob, Christian Weingart, Ellen Freiberger, Cornel Sieber, Johan Ärnlöv, Axel Carlsson, Tobias Feldreich

**Affiliations:** 1 Italian National Research Center on Aging (IRCCS INRCA), Ancona, Fermo and Cosenza, Italy; 2 Laboratory of Geriatric Pharmacoepidemiology and Biostatistics, IRCCS INRCA, Via S. Margherita 5, 60124 Ancona, Italy; 3 Geriatric Unit, Internal Medicine Department, Bellvitge University Hospital – IDIBELL – L’Hospitalet de Llobregat, Barcelona, Spain; 4 Section of Geriatric Medicine, Department of Internal Medicine, Erasmus MC, University Medical Center Rotterdam, Rotterdam, The Netherlands; 5 Department of Internal Medicine, Medical University of Graz, Graz, Austria; 6 Department of Medical Sciences, Uppsala University, Uppsala, Sweden; 7 School of Health and Social Studies, Dalarna University, Falun, Sweden; 8 Division of Family Medicine, Department of Neurobiology, Care Sciences and Society, Karolinska Institutet, Huddinge, Sweden; 9 Department of General Internal Medicine and Geriatrics, Institute for Biomedicine of Aging, Krankenhaus Barmherzige Brüder, Friedrich-Alexander-Universität Erlangen-Nürnberg, Regensburg, 93049 Germany; 10 Department of Internal Medicine-Geriatrics, Institute for Biomedicine of Aging, Krankenhaus Barmherzige Brüder, Friedrich-Alexander Universität Erlangen-Nürnberg, Koberger Strasse 60, 90408 Nuremberg, Germany; 11 Department of Geriatrics, Healthy Ageing Research Centre, Medical University of Lodz, Lodz, Poland; 12 Department of Geriatric Medicine, Hospital Clinico San Carlos, Madrid, Spain; 13 The Recanati School for Community Health Professions at the faculty of Health Sciences, Ben-Gurion University of the Negev, Beersheba, Israel; 14 Maccabi Health Organization, Negev District, Israel

**Keywords:** Chronic kidney disease, Multimorbidity, Short physical performance battery, Older

## Abstract

**Background:**

Chronic kidney disease (CKD) is known to be associated with several co-occurring conditions. We aimed at exploring multimorbidity patterns associated with CKD, as well as the impact of physical performance and CKD severity on them in a population of older outpatients.

**Methods:**

Our series consisted of 2252 patients enrolled in the Screening of CKD among Older People across Europe multicenter observational study. Hypertension, stroke, transient ischemic attack, cancer, hip fracture, osteoporosis, Parkinson’s disease, asthma, chronic obstructive pulmonary disease, congestive heart failure, angina, myocardial infarction, atrial fibrillation, anemia, CKD (defined as GFR < 60, < 45 or < 30 ml/min/1.73 m^2^), cognitive impairment, depression, hearing impairment and vision impairment were included in the analyses. Physical performance was assessed by the Short Physical Performance Battery (SPPB) and used as stratification variable. Pairs of co-occurring diseases were analyzed by logistic regression. Patterns of multimorbidity were investigated by hierarchical cluster analysis.

**Results:**

CKD was among the most frequently observed conditions and it was rarely observed without any other co-occurring disease. CKD was significantly associated with hypertension, anemia, heart failure, atrial fibrillation, myocardial infarction and hip fracture. When stratifying by SPPB, CKD was also significantly associated with vision impairment in SPPB = 5–8 group, and hearing impairment in SPPB = 0–4 group. Cluster analysis individuated two main clusters, one including CKD, hypertension and sensory impairments, and the second including all other conditions. Stratifying by SPPB, CKD contribute to a cluster including diabetes, anemia, osteoporosis, hypertension and sensory impairments in the SPPB = 0–4 group. When defining CKD as eGFR< 45 or 30 ml/min/1.73 m^2^, the strength of the association of CKD with hypertension, sensory impairments, osteoporosis, anemia and CHF increased together with CKD severity in pairs analysis. Severe CKD (eGFR< 30 ml/min/1.73 m^2^) contributed to a wide cluster including cardiovascular, respiratory and neurologic diseases, as well as osteoporosis, hip fracture and cancer.

**Conclusions:**

CKD and its severity may contribute significantly to specific multimorbidity patterns, at least based on the cluster analysis. Physical performance as assessed by SPPB may be associated with not negligible changes in both co-occurring pairs and multimorbidity clusters.

**Trial registration:**

The SCOPE study is registered at clinicaltrials.gov (NCT02691546).

## Background

The progressive aging of the population in industrialized countries is accompanied by a dramatic increase in the prevalence of multiple chronic diseases [1]. Traditionally, research has embraced the comorbidity conceptual framework, i.e. a framework which identifies an index disease and focuses on the probability of having other (secondary) diseases [2]. In an attempt to meet the complex problems posed by older patients with multiple chronic diseases, the attention has progressively shifted from the comorbidity to the multimorbidity conceptual framework. In this approach the co-occurrence of two or more diseases is taken into consideration without identifying an index disease [3, 4]. From the clinical point of view, the multimorbidity concept tends to push geriatric medicine towards an individual-oriented and no longer disease-oriented approach. Several different analytic approaches can be used to investigate multimorbidity. Studies explored multimorbidity by applying different methods (e.g. prevalence figures, conditional count, logistic regression models, cluster or network analysis and data mining techniques) to disease variables in different populations, and provided convincing evidence that chronic diseases may combine each other not simply due to chance [5–10].

Chronic kidney disease (CKD) is among the most frequent chronic conditions observed among older patients, and it dramatically impacts health status and survival in the general population, as well as in older populations [11–14]. Although CKD is known to be associated with several long lasting conditions, such as hypertension, diabetes, heart failure, anemia and osteoporosis, studies investigating patterns of multimorbidity including CKD provided heterogeneous results. Formiga et al. [6] showed that CKD may contribute to a multimorbidity cluster including cardio- and cerebro-vascular disease, atrial fibrillation, diabetes mellitus and visual impairment. More recently, CKD was found to contribute to a larger cluster including cardiovascular diseases (excluded coronary artery disease), endocrine abnormalities, neurological disorders including dementia, respiratory and muscle-skeletal diseases, infections and sensory impairment [10]. Thus, multimorbidity patterns involving CKD are worth of testing in other different populations.

Additionally, data about the impact of potentially relevant stratification variables on patterns of multimorbidity are very limited. Current evidence suggests that age, sex, and race/ethnicity may be associated with small but not negligible changes in multimorbidity patterns [10]. However, whether patterns of multimorbidity may change as a function of physical performance has not been investigated until now, despite the bidirectional interplay of multimorbidity and functional decline is more and more recognized as a crucial step in the assessment of health and care needs of older complex patients [15].

Finally, CKD severity may also affect multimorbidity patterns. Indeed, many of CKD-related concordant comorbidities, such as anemia, osteoporosis and heart failure, are known to be associated with the severity of CKD [16].

By including a detailed collection of data about diagnoses and comprehensive geriatric assessment including short physical performance battery (SPPB) [17], the Screening for CKD among Older People across Europe (SCOPE) study represents a valuable opportunity to explore multimorbidity patterns as well as the impact of physical performance and CKD severity on them in a population of older community-dwelling people. These were the main objectives of our study.

## Methods

The SCOPE study (European Grant Agreement no. 436849), is a multicenter 2-year prospective cohort study involving patients older than 75 years attending geriatric and nephrology outpatient services in participating institutions in Austria, Germany, Israel, Italy, the Netherlands, Poland and Spain. Methods of the SCOPE study have been extensively described elsewhere [18]. Briefly, all patients attending the outpatient services (7 geriatric and internal medicine and 2 nephrology services) at participating centers from August 2016 to August 2018 were asked to participate. Only patients signing a written informed consent entered the study. Age greater or equal to 75 years was the only inclusion criteria, the exclusion criteria were: end-stage renal disease or dialysis at time of enrollment; history of solid organ or bone marrow transplantation; active malignancy within 24 months prior to screening or metastatic cancer; life expectancy less than 6 months (based on the judgment of the study physician after careful medical history collection and diagnoses emerging from examination of clinical documentation exhibited); severe cognitive impairment (Mini Mental State Examination < 10); any medical or other reason (e.g. known or suspected patients’ inability to comply with the protocol procedure) in the judgement of the investigators, that the patient was unsuitable for the study; unwilling to provide consent and limited possibility to attend follow-up visits. The study protocol was approved by ethics committees at all participating institutions, and complies with the Declaration of Helsinki and Good Clinical Practice Guidelines. Only baseline data are used in the present study.

Overall, 2461 patients were initially enrolled in the study. Of them, 209 patients were excluded from this study because of incomplete baseline data, thus leaving a final sample of 2252 patients to be included in the analysis.

### Study variables

Diagnoses were ascertained by the study physician by clinical history and physical examination. Physicians asked the participants to show all clinical records and prescription forms or drug containers of the drugs used. The following diagnoses were included in the analysis: hypertension, stroke, transient ischemic attack (TIA), cancer, hip fracture, osteoporosis, Parkinson’s disease, asthma, chronic obstructive pulmonary disease (COPD), congestive heart failure (CHF), angina, myocardial infarction, atrial fibrillation. Chronic kidney disease was defined on the basis of estimated glomerular filtration rate (eGFR) < 60 ml/min/1.73 m^2^. eGFR was calculated by creatinine-based Berlin Initiative Study (BIS) equation [19]. Such equation has been developed to be used in older people and has been externally validated against gold-standard measured GFR [20, 21]. Cognitive impairment was defined as age- and education-adjusted Mini Mental State Examination (MMSE) score less than 24 [22]. Depression was ascertained on the basis of Geriatric Depression Scale (GDS) score greater than 5 [23]. Vision and hearing impairment were coded on a scale from 0 (adequate) to 4 (no vision/hearing present) [24], and patients with at least mild deficit (scoring 1 or more) were considered as impaired. Anemia was defined as a hemoglobin level less than 13 g/dL in men and less than 12 g/dL in women. Multimorbidity was defined as the co-occurrence of two or more diseases.

Further variables included in the analysis were age, sex, and physical performance. Physical performance was measured by SPPB [17]. The SPPB includes gait speed (usual time to walk 4 m), five chair-stands test (time to rise from a chair and return to the seated position five times without using arms), and balance test (ability to stand with the feet together in the side-by-side, semi-tandem, and tandem positions). A score from 0 to 4 was assigned to performance on each task. Individuals received a score of 0 for each task they were unable to complete. Summing the three individual categorical scores, a summary performance score was created for each participant (range, 0–12), with higher scores indicating better lower body function. SPPB score was stratified using the following cut-offs: 9–12, best performance; 5–8, intermediate performance; 0–4, worst performance [17].

### Analytic approach

First, descriptive analysis of the study population grouped according to SPPB score was provided. Data were reported as mean ± SD for continuous variables and number (percentage) for categorical ones. Chi-square test was used to analyze categorical variables, while ANOVA one way was used for continuous ones. For each chronic disease included in the analysis, its prevalence with and without co-occurring disease(s) was estimated. The expected prevalence of disease pairs involving CKD was computed and compared with the observed co-prevalence. To confirm the results of the pairs analysis and to control for possible confounders, logistic regression models were conducted to analyze the association between each pair of co-occurring diseases. This analysis was adjusted for age, sex, and all of the other diseases. To analyze different patterns of associative multimorbidity, without any a priori hypothesis, we used a hierarchical agglomerative clustering approach to create dendrograms. A binary distance measure (squared euclidean distance) was used to produce the distance matrix resulting in more distinctive clusters compared with other proximity measures. For dendrograms analysis we used the Ward method as previously reported [10] to find compact clusters and minimizes the variance within clusters [25]. In order to investigate the impact of physical performance on multimorbidity patterns, analyses were repeated after stratification by SPPB score. Finally, in order to investigate the impact of CKD severity on patterns of multimorbidity, disease pairs and cluster analyses were repeated by using two additional eGFR thresholds, < 45 and < 30 ml/min/1.73 m^2^, to define CKD.

Statistical analysis was carried out by using SPSS for Win V23.0 statistical software package (SPSS Inc., Chicago, IL, USA).

## Results

### Descriptive analysis

General characteristics of the study population are reported in Table 1. Age ranged between 75 and 96 years, and more than half of enrolled patients were women. CKD was among the most frequently observed conditions (66.0%), together with hypertension and sensory impairments (Table 1). CKD occurred without any other co-occurring disease in only 0.9% of participants. The corresponding figures for other diagnoses were 1.5% for hypertension, 1% for vision impairment, 0.4% for hearing impairment. All other diagnoses were also rarely present without any associated disease.

**Table 1:** General characteristics and prevalence of selected diseases among SCOPE study participants (*N* = 2252) and their distribution across physical performance status

	All patients *N* = 2252	SPPB score			*P*
		9–12 *N* = 1391	5–8 *N* = 614	0–4 *N* = 247	
Age, years	80.3 ± 4.1	79.6 ± 3.7	81.2 ± 4.4	82.4 ± 4.7	< 0.001
Sex, women	1254 (55.7)	698 (50.2)	381 (62.1)	175 (70.9)	< 0.001
Hypertension	1729 (76.8)	997 (71.7)	509 (82.9)	223 (90.3)	< 0.001
CKD	1487 (66.0)	871 (62.6)	415 (67.6)	201 (81.4)	< 0.001
Vision impairment	1299 (57.7)	758 (54.5)	370 (60.3)	171 (69.2)	< 0.001
Hearing impairment	1165 (51.7)	661 (47.5)	348 (56.7)	156 (63.2)	< 0.001
Osteoporosis	687 (30.5)	324 (23.3)	243 (39.6)	120 (48.6)	< 0.001
Diabetes	566 (25.1)	280 (20.1)	188 (30.6)	98 (39.7)	< 0.001
Anemia	476 (21.1)	228 (16.4)	150 (24.4)	98 (39.7)	< 0.001
Cancer	388 (17.2)	234 (16.8)	104 (16.9)	50 (20.2)	0.413
CHF	373 (16.6)	187 (13.4)	119 (19.4)	67 (27.1)	< 0.001
Atrial fibrillation	344 (15.3)	159 (11.4)	116 (18.9)	69 (27.9)	< 0.001
Depression	316 (14.0)	134 (9.6)	113 (18.4)	69 (27.9)	< 0.001
COPD	266 (11.8)	149 (10.7)	86 (14)	31 (12.6)	0.101
Myocardial infarction	217 (9.6)	112 (8.1)	65 (10.6)	40 (16.2)	< 0.001
TIA	196 (8.7)	104 (7.5)	64 (10.4)	28 (11.3)	0.029
Cognitive impairment	165 (7.3)	71 (5.1)	59 (9.6)	35 (14.2)	< 0.001
Angina	133 (5.9)	72 (5.2)	39 (6.4)	22 (8.9)	0.062
Stroke	131 (5.8)	59 (4.2)	45 (7.3)	27 (10.9)	< 0.001
Asthma	124 (5.5)	73 (5.2)	30 (4.9)	21 (8.5)	0.087
Hip fracture	111 (4.9)	32 (2.3)	51 (8.3)	28 (11.3)	< 0.001
Parkinson	45 (2.0)	11 (0.8)	17 (2.8)	17 (6.9)	< 0.001

Data are mean ± SD or number of cases (percentage)

When stratifying population by physical performance, SPPB = 9–12 was observed in 1391(61.8%) patients, while 614 patients (27.3%) had SPPB = 5–8, and 247 patients (11.0%) had SPPB = 0–4. This latter group included only a minority of patients (*n* = 24) not able to do any SPPB tasks and scoring 0 at physical performance assessment. Patients with lower SPPB scores were older and more frequently women (Table 1). Additionally, almost all diagnoses were more prevalent among physically impaired patients (Table 1).

### Patterns of multimorbdity and physical performance

Observed and expected prevalence of the most frequently co-occurring pairs of diseases and their association are shown in Table 2. The most frequent co-occurring pairs involving CKD were those with hypertension, sensory impairments, osteoporosis, diabetes and anemia. After adjusting for age, sex and all other diseases included in the analyses, CKD was significantly associated with hypertension, anemia, CHF, atrial fibrillation, myocardial infarction and hip fracture, while near significant associations were observed with hearing impairment, diabetes and cancer (Table 2). It is worth noting that 89.4% of diabetic patients were also affected by hypertension, and the association between CKD and diabetes became significant when removing hypertension from the fully adjusted model (OR = 1.36, 95%CI = 1.08–1.71).

**Table 2:** Observed and expected prevalence of co-occurring pairs of diseases involving CKD in the whole study population (*N* = 2252)

Co-occurring pairs		Cases n	Observed %	Expected %	Obs/Exp ratio	Adjusted for age and sex		Fully adjusted	
						OR	95%CI	OR	95%CI
CKD	Hypertension	1224	54.4	50.7	1.07	2.30	1.87–2.82	1.90	1.53–2.36
CKD	Vision impairment	880	39.1	38.1	1.03	1.25	1.04–1.50	1.06	0.87–1.29
CKD	Hearing impairment	822	36.5	34.2	1.07	1.37	1.15–1.64	1.20	0.98–1.46
CKD	Osteoporosis	449	19.9	20.1	0.99	1.02	0.83–1.25	0.95	0.76–1.18
CKD	Diabetes	420	18.7	16.6	1.12	1.63	1.31–2.03	1.22	0.97–1.55
Anemia	CKD	388	17.2	13.9	1.24	2.26	1.75–2.92	1.95	1.49–2.54
CHF	CKD	305	13.5	10.9	1.24	2.47	1.86–3.29	1.73	1.27–2.36
Cancer	CKD	282	12.5	11.4	1.10	1.36	1.06–1.75	1.27	0.98–1.64
Atrial fibrillation	CKD	276	12.3	10.1	1.22	2.07	1.55–2.75	1.57	1.15–2.13
CKD	Depression	212	9.4	9.3	1.02	1.06	0.81–1.37	0.93	0.71–1.23
CKD	COPD	202	9.0	7.8	1.15	1.58	1.16–2.14	1.26	0.91–1.74
CKD	Myocardial infarction	182	8.1	6.4	1.27	2.46	1.68–3.61	1.75	1.17–2.62
CKD	TIA	136	6.0	5.7	1.05	1.07	0.77–1.49	0.88	0.62–1.24
CKD	Cognitive impairment	120	5.3	4.8	1.10	1.15	0.80–1.67	1.06	0.72–1.56
CKD	Stroke	98	4.4	3.8	1.13	1.37	0.90–2.07	1.11	0.72–1.72
Angina	CKD	94	4.2	3.9	1.07	1.17	0.79–1.74	0.72	0.46–1.11
Asthma	CKD	81	3.6	3.6	0.99	1.03	0.69–1.52	0.85	0.56–1.28
CKD	Hip fracture	65	2.9	3.3	0.89	0.57	0.38–0.86	0.59	0.38–0.91
CKD	Parkinson	31	1.4	1.3	1.04	0.94	0.49–1.82	0.83	0.42–1.67

When stratifying pairs analysis by physical performance, CKD was significantly associated with hypertension (OR = 1.66, 95%CI = 1.28–2.14), CHF (OR = 1.63, 95%CI = 1.09–2.43), atrial fibrillation (OR = 1.82, 95%CI = 1.18–2.79) and hip fracture (OR = 0.34, 95%CI = 0.15–0.76) among patients with SPPB score = 9–12. At variance, the significant co-occurring pairs involving CKD were those with hypertension (OR = 2.79, 95%CI = 1.72–4.52), CHF (OR = 2.39, 95%CI = 1.27–4.47), anemia (OR = 3.68, 95%CI = 2.09–6.48) and vision impairment (OR = 1.50, 95%CI = 1.02–2.20) among patients with SPPB = 5–8. Finally, significant co-occurring pairs among patients with SPPB = 0–4 were those involving hypertension (OR = 3.54, 95%CI = 1.21–10.4), anemia (OR = 2.10, 95%CI = 1.03–4.68) and hearing impairment (OR = 2.20, 95%CI = 1.02–4.93).

**Fig. 1:**
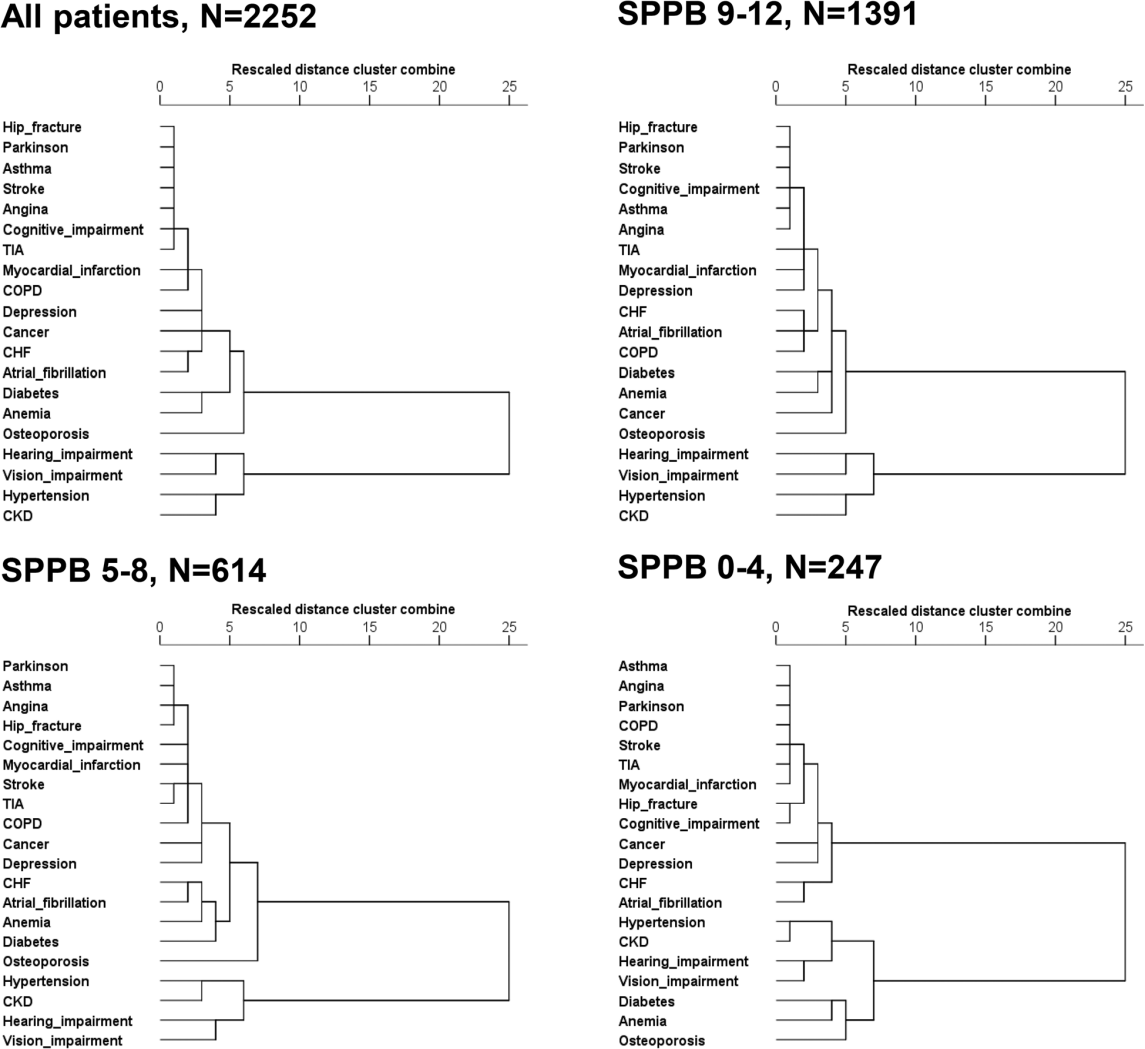
Dendrograms resulting from clusters analysis testing the distribution and aggregation of chronic diseases in the whole study population and after stratifying by SPPB

Finally, patterns of multimorbidity from cluster analysis are reported in Fig. 1. Overall, cluster analysis individuated two main clusters, one including CKD, hypertension and sensory impairments, and the second including all other conditions. When stratifying this analysis by SPPB, these patterns were substantially confirmed among patients with SPPB = 9–12 or SPPB = 5–8. At variance, the pattern of multimorbidity involving CKD was clearly different among patients with lowest level of physical performance where a sub-cluster including diabetes, anemia and osteoporosis appeared near to the one including hypertension, CKD and sensory impairments. When repeating this latter analysis after excluding 24 patients scoring 0 at SPPB assessment, the dendrogram remained unchanged (not shown).

### Patterns of multimorbidity and CKD severity

**Fig. 2:**
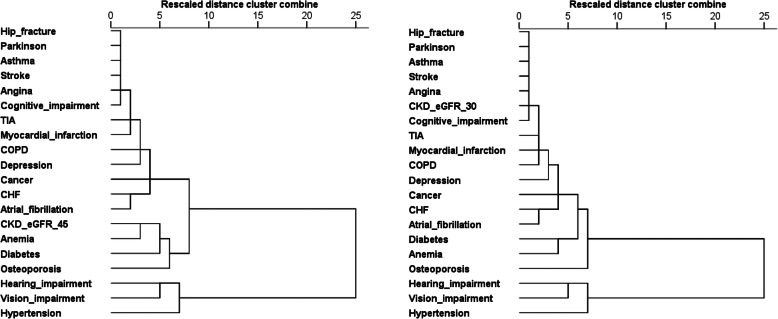
Dendrograms resulting from clusters analysis testing the distribution and aggregation of chronic diseases in the study population using two different eGFR threshold to define CKD (< 45 or < 30 ml/min/1.73 m^2^)

When defining CKD as eGFR< 45 or 30 ml/min/1.73 m^2^, the strength of the association of CKD with hypertension, sensory impairments, osteoporosis, anemia and CHF increased together with CKD severity (Table 3). Finally, patterns of multimorbidity also changed significantly in relation to CKD severity. Indeed, eGFR< 45 ml/min/1.73 m^2^ contributed to a sub-cluster including anemia, diabetes and osteoporosis, while severe CKD defined as eGFR< 30 ml/min/1.73 m^2^ contributed to a wide cluster including cardiovascular, respiratory and neurologic diseases, as well as osteoporosis, hip fracture and cancer Fig. 2.

**Table 3:** Observed and expected prevalence of co-occurring pairs of diseases involving CKD defined as eGFR< 45 (upper panel) or < 30 ml/min/1.73 m^2^ (lower panel)

Co-occurring pairs		Cases n	Observed %	Expected %	Obs/Exp ratio	Adjusted for age and sex		Fully adjusted	
						OR	95%CI	OR	95%CI
***CKD = eGFR < 45 ml/min/1.73 m***^***2***^									
CKD	Hypertension	509	22.6	19.0	1.2	3.87	2.82–5.30	3.01	2.14–4.20
CKD	Vision impairment	359	15.9	14.3	1.1	1.56	1.26–1.91	1.21	0.95–1.53
CKD	Hearing impairment	360	16.0	12.8	1.2	1.79	1.46–2.19	1.48	1.17–1.87
CKD	Osteoporosis	186	8.3	7.6	1.1	1.38	1.10–1.73	1.22	0.94–1.56
CKD	Diabetes	211	9.4	6.2	1.5	2.31	1.86–2.86	1.62	1.27–2.04
Anemia	CKD	245	10.9	5.2	2.1	4.21	3.36–5.26	3.64	2.86–4.61
CHF	CKD	149	6.6	4.1	1.6	2.21	1.73–2.81	1.34	1.00–1.79
Cancer	CKD	121	5.4	4.3	1.3	1.36	1.06–1.74	1.21	0.92–1.58
Atrial fibrillation	CKD	124	5.5	3.8	1.5	1.64	1.27–2.11	1.19	0.88–1.59
CKD	Depression	78	3.5	3.5	1.0	0.99	0.74–1.32	0.79	0.57–1.08
CKD	COPD	95	4.2	2.9	1.4	1.65	1.24–2.18	1.31	0.95–1.80
CKD	Myocardial infarction	107	4.8	2.4	2.0	2.89	2.14–3.89	2.00	1.43–2.78
CKD	TIA	58	2.6	2.2	1.2	1.15	0.82–1.61	0.95	0.65–1.39
CKD	Cognitive impairment	46	2.0	1.8	1.1	0.98	0.67–1.41	0.90	0.60–1.35
CKD	Stroke	46	2.0	1.4	1.4	1.49	1.01–2.18	1.21	0.78–1.85
Angina	CKD	49	2.2	1.5	1.5	1.74	1.19–2.55	0.98	0.62–1.52
Asthma	CKD	32	1.4	1.4	1.0	1.15	0.74–1.76	0.93	0.58–1.49
CKD	Hip fracture	24	1.1	1.2	0.9	0.66	0.40–1.06	0.67	0.39–1.15
CKD	Parkinson	14	0.6	0.5	1.2	1.15	0.59–2.22	0.80	0.38–1.66
***CKD = eGFR < 30 ml/min/1.73 m***^***2***^									
CKD	Hypertension	135	6.0	4.8	1.3	8.44	3.43–20.7	5.73	2.29–14.3
CKD	Vision impairment	107	4.8	3.6	1.3	2.71	1.81–4.06	1.79	1.13–2.81
CKD	Hearing impairment	103	4.6	3.2	1.4	2.49	1.68–3.66	1.69	1.09–2.61
CKD	Osteoporosis	64	2.8	1.9	1.5	2.79	1.90–4.08	2.22	1.46–3.34
CKD	Diabetes	51	2.3	1.6	1.5	1.69	1.17–2.42	1.16	0.77–1.71
Anemia	CKD	83	3.7	1.3	2.8	5.53	3.83–7.96	4.64	3.16–6.79
CHF	CKD	49	2.2	1.0	2.1	2.70	1.86–3.91	1.77	1.14–2.73
Cancer	CKD	34	1.5	1.1	1.4	1.45	0.96–2.17	1.26	0.81–1.93
Atrial fibrillation	CKD	32	1.4	0.9	1.5	1.46	0.95–2.22	0.96	0.59–1.55
CKD	Depression	19	0.8	0.9	0.9	1.00	0.60–1.65	0.81	0.46–1.38
CKD	COPD	25	1.1	0.7	1.5	1.45	0.91–2.30	0.97	0.56–1.65
CKD	Myocardial infarction	28	1.2	0.6	2.0	2.05	1.30–3.21	1.49	0.89–2.50
CKD	TIA	11	0.5	0.5	0.9	0.78	0.40–1.47	0.64	0.31–1.28
CKD	Cognitive impairment	7	0.3	0.5	0.7	0.57	0.25–1.25	0.54	0.23–1.23
CKD	Stroke	13	0.6	0.4	1.7	1.52	0.82–2.78	1.35	0.68–2.65
Angina	CKD	8	0.4	0.4	1.1	0.88	0.41–1.84	0.46	0.20–1.03
Asthma	CKD	7	0.3	0.3	0.9	0.99	0.44–2.16	0.87	0.38–1.99
CKD	Hip fracture	7	0.3	0.3	1.0	0.93	0.41–2.07	0.83	0.34–1.98
CKD	Parkinson	4	0.2	0.1	1.6	1.27	0.44–3.63	0.85	0.27–2.63

## Discussion

Findings from the present study suggests that CKD significantly contributes to multimorbidity patterns in a population of older outpatients. Additionally, simple count of disease may hide several important information otherwise obtained by the analysis of most relevant pairs of co-occurring disease or disease clusters. Indeed, as already reported in former studies [5, 6, 26, 27] the occurrence of individual conditions without any associated disease was rarely observed in our study population. Finally, and in a relevant way, our study also suggests that multimorbidity patterns may change as a function of physical performance as assessed by SPPB.

The most relevant co-occurring pairs involving CKD observed in our study where those with hypertension, anemia, CHF, atrial fibrillation, myocardial infarction, hip fracture, and to a lesser extent hearing impairment, diabetes and cancer. The weak association between CKD and diabetes was not surprising given that almost all diabetic patients also had high blood pressure in our study and removing hypertension from the multivariable model made statistically significant such association in pairs analysis. Additionally, it is worth noting that after stratification by SPPB score other significant pairs emerged among patients with SPPB = 5–8 (vision impairment) and SPPB = 0–4 (hearing impairment). Thus, it seems that a simple physical performance test, already known to predict mobility disability, ADL dependency, rehospitalization and death [28–31], may also help to describe patients with somewhat different profiles of co-occurring pairs of diseases.

While the association between CKD and cardiovascular diseases, anemia, diabetes and mineral bone disease (including fractures) is well recognized and extensively addressed in clinical guidelines where specific recommendations are provided for these conditions [32], the combination of CKD with cancer and sensory impairments deserves to be discussed. Chronic kidney disease (CKD) and cancer are strictly related each other. Cancer can cause CKD either directly or indirectly through the adverse effects of treatments. Conversely, CKD may represent a relevant risk factor for cancer [33]. Indeed, though the increased risk of death observed among CKD patients is mainly attributable to cardiovascular disease, the incidence of cancer is also increased [16]. Cancer can affect the kidney either as glomerular lesions or as a result of the toxic effects of medication or radiation with acute (thrombotic microangiopathy, acute kidney injury, interstitial nephropathies among others) or chronic processes (worsening of CKD after nephrectomy due to renal cancer, interstitial fibrosis, water and electrolytes disorders) [34]. It is worth noting that we only considered history of cancer in our study, while patients carrying active malignancies during the last 24 months before enrollment were excluded. Nevertheless, older cancer survivors may be more likely to develop frailty or worsening of the health status, and such association may be especially relevant among individuals with a recent (< 10 years) history of cancer [35].

Given the mean age of enrolled patients, the association between CKD and sensory impairment may be simply related to older age independent of kidney function. However, several potential mechanisms may underly such association. Hearing loss was found to be highly prevalent among CKD patients [36, 37], and increasing serum creatinine or blood pressure was found associated with increasing hearing threshold [38]. Several similarities between cochlea and kidney can be observed, including membranous structures, the central role of ciliated epithelial cells, and tubular organization [39]. In experimental uremic animals, a significant decrease in cochlear Na + −K + -ATPase activity resulted in hearing impairment [40]. Additionally, ototoxic medications may also contribute to the observed association. For example, furosemide may affect ionic gradients between the endolymph and perilymph thus altering endocochlear potential and leading to cochlear dysfunction [41]. The association between CKD and vision impairment was also reported to be highly prevalent in CKD patients. A population study involving 10,033 adults aged 40–80 years showed that the prevalence of vision impairment and ocular disease were significantly higher in participants with (36.1 and 84.7%) compared to those without CKD (12.9 and 54.3%, both *p* <  0.001) [42]. Diabetic retinopathy and age-related macular degeneration are most consistently associated with CKD [43]. It is worth noting that mechanisms underlying CKD, such as vascular remodeling, endothelial dysfunction, atherosclerosis, inflammation, and oxidative stress are also involved in the development of many eye diseases. Besides diabetes and hypertension, metabolic disorders associated with CKD, such as oxidative stress, uremia, anemia, as well as specific treatment, such as steroids and dialysis may be also involved [43]. Despite this bulk of evidence, screening for vision and hearing impairment is not currently recommended in CKD clinical guidelines and our findings suggest for its potential usefulness in this vulnerable population.

Cluster analysis provided quietly consistent results showing that CKD may contribute to a cluster of multimorbidity including hypertension and sensory impairments in the whole study population. These findings are somewhat different from those reported in former studies and characteristics of population, as well as the list of diagnoses included in the analysis likely account for these differences. Marengoni et al. identified five major clusters (two linked to vascular diseases with hypertension and heart failure playing the main role and the others to dementia, diabetes mellitus, and malignancy) in a population of 1099 community-dwelling people aged 77 or more. However, CKD was not included in the list of diagnoses [5]. In the study by Formiga et al. [6], the list of diagnoses was very similar to that in our study and four main clusters were identified in a population of 328 oldest old people aged 85 or more. CKD contributed to a multimorbidity cluster including atrial fibrillation, heart failure, visual impairment, stroke, hypertension and diabetes mellitus [6]. More recently, Zemedikun et al. [10] identified 3 main multimorbidity clusters with CKD contributing to the wide cluster including cardiovascular diseases (excluded coronary artery disease), endocrine abnormalities, neurological disorders including dementia, respiratory and muscle-skeletal diseases, infections and sensory impairment. However, at variance from our study their analysis was carried out in a population younger than 70 years [10].

Interestingly, when stratifying analysis according to physical performance, the cluster including CKD was “enriched” by a sub-cluster including anemia and osteoporosis (i.e. two well-known complications of CKD, namely CKD-related anemia and CKD-Mineral Bone Disease (CKD-MBD)) and diabetes (i.e. a leading cause of CKD) among patients with lowest SPPB scores. Thus, both co-occurring pairs and clustering of diseases may change as a function of SPPB among older people in the present study, and SPPB is among the most useful frailty tools for the identification of patients that may benefit from interventions aimed at improving functional capacity in primary care settings [44]. Our findings are in keeping with current guidelines stating that frailty and sensory impairments are very relevant items when investigating multimorbidity, while defining multimorbidity by simple counts of health conditions may be misleading [45]. Indeed, current evidence suggests that frailty and multimorbidity are strictly related each other in older adults [46, 47]. Additionally, physical performance assessed by gait speed and grip strength was recently found significantly associated with both the development of multimorbidity and worsening pre-existing multimorbidity, with evidence of a dose–response relationship [48]. Our results further strengthen the need to assess physical function in clinical practice and to establish specific function-oriented interventions able to reduce the burden of multimorbidity and related negative outcomes.

Finally, this is the first study including CKD severity in the analysis of multimorbidity patterns. Indeed, CKD severity was not considered in former multimorbidity studies [6, 10]. Besides confirming that the strength of the association between CKD and selected complications, i.e. hypertension, sensory impairments, osteoporosis, anemia and CHF may increase together with CKD severity [16], our study adds to current knowledge by showing that the complexity of multimorbidity cluster including CKD may also change in relation to CKD severity. These findings suggest that severity of individual diseases should be taken into consideration in future studies of multimorbidity among older complex patients.

Limitations of our study deserve to be mentioned. The cross-sectional design did not allow to investigate pathways leading to multimorbidity and the prognostic impact of specific multimorbidity patterns. Additionally, the list of diagnoses included in the analysis could not be considered exhaustive. However, we used a list of diagnoses very similar to that already used in studies investigating multimorbidity patterns. Nevertheless, the prevalence of individual diseases and that of individual diagnoses with multimorbidity was somewhat higher in the SCOPE study population compared to former studies [5, 6, 10], likely because we enrolled older people attending outpatients services in participating institutions with an extensive assessment and a complete analysis of clinical documentation exhibited by patients and caregivers, while former studies were population-based [5, 6, 10]. Our study did not include the assessment of severity of each individual disease other than CKD and findings from CKD severity analysis suggest that disease severity may represent a relevant confounder. The very rare occurrence of selected diagnoses without multimorbidity prevented us to explore differences between patients with and without multimorbidity. Finally, we need to recognize that while the analysis of co-occurring pairs may have an immediate clinical relevance, results obtained by hierarchical cluster analysis of diagnoses is less easy to be translated to clinical practice. Nevertheless, these exploratory data may represent a sound scientific basis for future studies on the clinical characterization of patient clusters and/or groups carrying specific multimorbidity patterns involving CKD. This study also has important strengths, including the enrollment of a real-world European population of older community-dwelling people and the opportunity to investigate the impact of objectively measured physical performance on patterns of multimorbidity.

## Conclusions

CKD contributes significantly to multimorbidity in a population of older outpatients and it was rarely observed without any co-occurring disease. The most significant co-occurring pairs involving CKD included hypertension, anemia, CHF, atrial fibrillation, myocardial infarction, hip fracture, and to a lesser extent hearing impairment, diabetes and cancer. Cluster analysis showed that CKD may cluster with hypertension and sensory impairments. Physical performance as assessed by SPPB may be associated with not negligible changes in both co-occurring pairs and multimorbidity clusters. These findings strengthen the need of assessing physical performance and investigating interventions targeting physical function among complex multimorbid patients in an attempt to improve outcomes and reduce costs associated with multimorbidity. Finally, CKD severity may significantly affect patterns of multimorbidity, which suggests that disease severity should be further investigated in multimorbidity studies.

## Data Availability

The datasets generated and/or analysed during the current study are available in the SCOPE repository (www.scopeproject.eu).

## Abbreviations

ADL: Activities of Daily living
BIS: Berlin Initiative Study
CHF: Congestive heart failure
CKD: Chronic kidney disease
COPD: Chronic obstructive pulmonary disease
eGFR: Estimated glomerular filtration rate
SPPB: Short physical performance battery
TIA: Transient ischemic attack
